# Global, region and country burden of osteoarthritis at different sites in middle-aged and elderly populations from 1990 to 2021: a systematic analysis of the 2021 global burden of disease study

**DOI:** 10.3389/fmed.2025.1567303

**Published:** 2025-05-12

**Authors:** Guoxin Huang, Yiwen Zheng, Weimin Hong, Xiaohong Qu, Wentao Yang, Hui Cao, Fangtao Tian, Hongming Lin, Bin Pei, Bingqian Chen, Shu’e Yang, Da Qian

**Affiliations:** ^1^Department of Evidence-Based Medicine Center, Xiangyang No.1 People’s Hospital, Hubei University of Medicine, Xiangyang, China; ^2^Emergency and Critical Care Center, Intensive Care Unit, Zhejiang Provincial People’s Hospital, Affiliated People’s Hospital, Hangzhou Medical College, Hangzhou, China; ^3^Department of Pharmacy, The Third Affiliated Hospital (The Affiliated Luohu Hospital) of Shenzhen University, Shenzhen, China; ^4^Department of Orthopedic Surgery, Changshu Hospital Affiliated to Soochow University, Changshu No.1 People’s Hospital, Changshu, China; ^5^Department of Orthopedic Surgery, Xiangyang No.1 People’s Hospital, Hubei University of Medicine, Xiangyang, China; ^6^Xiangyang City Hospital for Tuberculosis, Xiangyang, China; ^7^Department of Burn and Plastic Surgery-Hand Surgery, Changshu Hospital Affiliated to Soochow University, Changshu No.1 People’s Hospital, Changshu, China

**Keywords:** Osteoarthritis, disease burden, decomposition analysis, health inequalities analysis, middle-aged and elderly people

## Abstract

**Objective:**

To explore the burden and trend of osteoarthritis (OA) at different sites in middle-aged and elderly people (45 years and older) from 1990 to 2021.

**Methods:**

Age-standardized incidence rates, prevalence rates, disability-adjusted life years (Daly) rates and average annual percent change were used to quantify the disease burden and trend of OA at different sites. Decomposition analysis was conducted to explore the impact of three population-level determinants on the burden of OA and the distribution of OA burden inequality in the Socio-Demographic Index (SDI) across countries.

**Results:**

The age-standardized prevalence rate had increased by 8.9%, and the OA cases had increased by 2.41 times compared to 1990. The incidence and prevalence of knee, hip and hand OA decreased sequentially, while high SDI regions tended to have higher age-standardized incidence rates, prevalence rates, and Daly rates. Decomposition analysis revealed that 85.9% of the increase in OA age-standardized Daly rates was attributable to population growth. This increase was most pronounced in high SDI populations for hip OA and middle SDI populations for knee and hand OA. From 1990 to 2021, the inequality in overall OA burden between countries had decreased. The absolute inequality gap for hand OA had narrowed the most significantly (45.3%), which followed by knee OA (11.9%), while the inequality gap for hip OA has slightly increased.

**Conclusion:**

In summary, all parts of the OA burden in middle-aged and elderly people had steadily increased from 1990 to 2021, which calls to implement personalized prevention targeting different parts of OA.

## 1 Introduction

Osteoarthritis (OA) is a chronic and disabling condition predominantly prevalent among middle-aged and older adults, characterized by degenerative changes and loss of cartilage, as well as reactive bone proliferation at the attachment sites of joint margins, ligaments, and subchondral bone, leading to osteophyte formation (1–3). With the acceleration of aging and increasing average life expectancy, OA has become a significant issue impacting the physical and mental health of the global elderly population (4). In a cohort study of Mexican Americans, it was found that patients with OA progressed to daily dysfunction 1.12-1.35 times faster than those without OA (5). In a national survey in South Korea, individuals with OA had a higher estimation of years lived with disability (6). In addition, studies have shown that OA is associated with increased rates of depressive symptoms and suicidal ideation (7, 8). Notably, the incidence of OA rises with age, experiencing a sharp increase between 40 and 50 years (9, 10). As OA progresses, the pathological changes become irreversible with advancing age. Consequently, elderly OA patients often present with late-stage disease compared to younger patients, with the treatment frequently involving joint replacement surgery, which imposes substantial economic burdens on both individuals and society (11). A systematic review of 54 studies showed that the range of costs directly associated with OA widened over time, but it did not result in improvements in health-related quality of life (12). Consequently, comprehending the disease burden of OA is essential for the development of effective prevention and management strategies targeting middle-aged and older individuals.

The manifestations of OA are highly variable, affecting various joints throughout the body. Historically, the knee and hip joints have been the most extensively studied (13). However, research on OA in other joints remains limited, despite the relatively high radiographic prevalence of hand OA (14). Due to the deviation of OA definition between different studies or the insufficiency of data itself, it is difficult to form a horizontal, intuitive comparison between OA in different parts, and such comparisons are also influenced by the influence of different ages, genders and regions (10).

The Global Burden of Disease Study (GBD) is a comprehensive and rigorous scientific endeavor that meticulously quantifies the prevalence of major diseases, risk factors, and intermediate clinical outcomes using highly standardized methods, which facilitates robust comparisons across different time periods, populations, and health issues. The GBD provides annual estimates from 1990 to the present day, covering 371 diseases and injuries, as well as 3,499 related clinical outcomes (sequelae) across 204 countries and territories, and subnational units in over 20 countries (15).

To provide a thorough assessment of the burden of OA, this study examines the incidence, prevalence, and disability-adjusted life years (Daly) associated with various types of OA among individuals aged 45 years and older. We analyzed the correlation between OA burden and the Socio-Demographic Index (SDI) across both regional and national levels. Furthermore, we explored the demographic and epidemiological factors that have contributed to changes in OA burden over the past three decades, offering valuable insights for the prevention, management, and treatment of OA in middle-aged and older adults.

## 2 Materials and methods

### 2.1 Overview of the GBD

The GBD study, conducted by the Institute for Health Metrics and Evaluation, provides a detailed and contemporary evaluation of health loss due to 371 diseases, injuries, and impairments, as well as 87 risk factors, across 204 countries and territories. These data are further stratified by age and sex to offer a nuanced understanding of health impacts (16). The methodologies utilized in the GBD 2021 have been thoroughly described in previous research publications (17). Additionally, the GBD 2021 study adheres to the Guidelines for Accurate and Transparent Health Estimates Reporting to ensure the reliability and transparency of its findings.

### 2.2 Definition of OA in middle-aged and elderly individuals

OA is the most common type of arthritis, marked by chronic inflammation, joint degeneration, and structural alterations (18, 19). The reference case definition for OA is radiologically confirmed symptomatic OA with Kellgren-Lawrence grades 2–4 (20, 21). Specifically, grade 2 symptomatic OA is defined by the presence of at least one osteophyte in the affected joint and pain for at least 1 month within the past year. Grades 3–4 symptomatic OA require both osteophytes and joint space narrowing, with grade 4 also involving deformity and pain for at least 1 month within the past year. In the GBD study, OA was estimated separately for four sites: total OA, knee OA, hip OA, and hand OA. The corresponding International Classification of Diseases, 10th Revision (ICD-10) codes are M16-M18.9 (OA), M16-M16.9 (hip OA), M17-M17.9 (knee OA), and M18-M18.9 (hand OA). Due to counting the prevalence of OA in multiple sites as a single case per person without considering site-specific correlations, the combined prevalence of OA in unique sites exceeded the total number of OA cases, which is referred to as total OA (22).

The incidence of OA rises with age, particularly showing a significant increase between the ages of 40 and 50 (5, 6). According to the classification criteria set by the World Health Organization, middle-aged and elderly individuals are generally defined as those over 45 years old, representing the stage after youth in human life, including middle age and old age (23). In this study, we adopt this definition (45 years and older) to describe the burden of OA among middle-aged and elderly populations (Supplementary methods).

### 2.3 Data processing and disease modeling

Data processing and disease modeling were performed in line with the official guidelines of the GBD 2021 study. The estimation process was conceptually divided into eight major steps: (1) identification and extraction of data sources; (2) adjustment of data; (3) estimation of prevalence and incidence for causes and sequelae using Descriptive Epidemiology Meta-Regression (DisMod-MR) 2.1 or alternative methods for specific cause groups; (4) estimation based on impairment; (5) distribution of severity; (6) application of disability weights (DWs); (7) adjustment for comorbid conditions; and (8) calculation of Years Lived with Disability (YLDs) by sequelae and causes. Further details specific to OA are provided in Supplementary material.

### 2.4 Measures

In this study, the metrics used to assess disease burden included prevalence, incidence, and Daly rates. Prevalence, expressed as cases per 100,000 population, was calculated by dividing the total number of cases (both incident and existing) by the population size. Incidence, expressed as cases per 100,000 individuals, was determined by dividing the number of new cases by the population size (Supplementary methods).

For the GBD 2021 study, Daly rates were calculated by first estimating cause-specific mortality and non-fatal health loss. Daly rates were subsequently computed for each year by combining Years of Life Lost (YLLs) and YLDs for each age-sex-location group. While OA belongs to the disease burden of non-fatal health problems and does not need to consider YLLs, then Daly rates value is equal to YLDs.

Ultimately, to more accurately assess the disease burden, we computed age-standardized incidence rates, age-standardized prevalence rates, and age-standardized Daly rates.

### 2.5 SDI

SDI serves as a composite indicator that encapsulates the social and economic factors influencing health outcomes in different locations. In the GBD 2021 study, SDI values were standardized by multiplying by 100, yielding a scale ranging from 0 to 100. Based on these values, the 204 countries and regions were divided into five SDI quintiles: low, low-middle, middle, high-middle, and high (Supplementary methods).

### 2.6 Annual average percentage change

The disease burden of OA is evaluated across various historical periods and sexes through the use of age-standardized incidence rates, prevalence rates, and Daly rates. Joinpoint regression analysis identifies temporal trends, with the AAPC serving as a key estimate. This analysis spans 204 countries and regions from 1990 to 2021. An increasing trend is indicated by an AAPC value and 95% confidence interval (CI) both greater than 0, while a decreasing trend is signified when both are less than 0. If neither condition is met, the burden is considered stable.

### 2.7 Decomposition analysis

To elucidate the factors contributing to the changes in Daly number between 1990 and 2021, a decomposition analysis was conducted to quantify the impact of three primary determinants: alterations in age structure, variations in population size, and epidemiological shifts. In this context, epidemiological shifts refer to the changes in age- and population-adjusted mortality and morbidity rates.

### 2.8 Cross-national social inequalities analysis

To evaluate the disparities in OA burden across countries, we employed the inequality slope index and the health concentration index, which quantify absolute and relative inequalities, respectively. Smoothing spline models were used to examine the relationship between country-level age-standardized Daly rates and the SDI, with heteroscedasticity addressed through weighted regression analysis. The health concentration index was derived by calculating the area under the Lorenz concentration curve, based on the distribution of age-standardized Daly rates and the population ranked by SDI.

## 3 Results

### 3.1 The global burden of OA at different sites

In 2021, the global prevalence of OA increased approximately 1.41-fold compared to 1990, with an age-standardized prevalence rate of 23,919.11 per 100,000 (95% UI, 20,796.73–27,142.93), representing an approximate 8.9% increased from 21,956.95 per 100,000 (95% UI, 19,120.65–24,919.91) in 1990. Additionally, the incidence of OA rose approximately 1.34-fold since 1990, with an age-standardized incidence rate of 1,591.95 per 100,000 (95% UI, 1,232.14–1,992.15), representing an increase of about 9.2% from 1,458.06 per 100,000 (95% UI, 1,132.30–1,828.48) in 1990. The age-standardized Daly rates also increased approximately 1.43-fold compared to 1990, with an age-standardized Daly rates of 841.28 per 100,000 (95% UI, 404.84–841.28), reflecting a 9.7% rise from 766.64 per 100,000 (95% UI, 370.10–1,545.44) in 1990. Supplementary Table 1 further elucidated the burden of OA at different anatomical sites. In 1990, the number of cases and incidence of knee OA, hand OA, and hip OA decreased sequentially. By 2021, this trend continued for all three types. The age-standardized prevalence, incidence, and Daly rates for hip OA were 1,389.15 per 100,000 (95% UI, 1,012.06–1,840.47), 61.69 (95% UI, 32.47–102.34), and 43.81 (95% UI, 20.30–89.03), respectively, reflecting increases of 5.7, 6.2, and 5.5% compared to 1990. The age-standardized prevalence, incidence, and Daly rates for knee OA were 14,741.08 (95% UI, 12,121.92–17,680.86), 1,050.31 (95% UI, 760.82–1,408.44), and 470.86 (95% UI, 224.34–928.27), showing increases of 8.4, 7.3, and 8.3%, respectively, since 1990. For hand OA, the age-standardized prevalence, incidence, and Daly rates were 7,811.95 (95% UI, 5,611.49–10,380.20), 352.56 (95% UI, 190.17–582.07), and 246.97 (95% UI, 110.78–509.67), representing increases of 14.8, 17.8, and 14.8%, respectively, from 1990.

### 3.2 The burden of OA across different SDI regions

**FIGURE 1:**
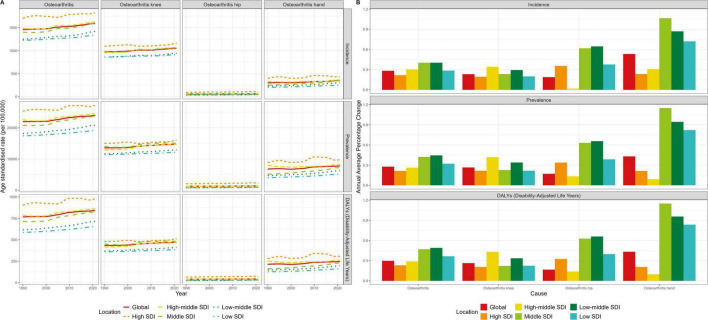
Age-standardized incidence rates and prevalence rates of different kinds of OA. **(A)** The trend chart of Daly rates in globally and different SDI. **(B)** The histogram of AAPC results.

Across the five SDI levels, high SDI regions tended to have higher age-standardized prevalence, incidence, and Daly rates of OA, whereas low SDI regions exhibited the opposite trend. The overall age-standardized prevalence, incidence, and Daly rates of OA decreased progressively from high to low SDI quintiles (Figure 1A). However, there were differences in the distribution of disease burden growth for various OA sites (Figure 1B). For knee OA, the high-middle and low-middle quintiles were slightly higher than other SDI levels. For hip OA, the middle and low-middle quintiles had significantly higher levels of burden growth than the others. For hand OA, the burden growth levels in the middle, low-middle and low quintiles were significantly higher than those in the other two quintiles, and decreased in the following order: middle, low-middle, low, high and high-middle quintile.

### 3.3 OA burden in different regions

Among the 21 global regions, the highest age-standardized prevalence, incidence, and Daly rates for the three types of OA were primarily found in East Asia, South Asia, Western Europe, and High-Income North America. Specifically, the age-standardized prevalence, incidence, and Daly rates for total OA were highest in the High-Income Asia Pacific region and lowest in Southeast Asia. For hip OA, these metrics were highest in High-Income North America and lowest in East, Southeast, and South Asia. Regarding knee OA, the highest rates were observed in the High-Income Asia Pacific, while the lowest rates were in Central Asia. For hand OA, the age-standardized prevalence and Daly rates were highest in Eastern Europe and lowest in East Asia, whereas the age-standardized incidence was highest in Central Asia and lowest in Oceania (Supplementary Tables 2-4).

### 3.4 OA burden in different countries

Among the 204 countries worldwide, the Republic of Korea exhibited the highest age-standardized prevalence, incidence, and Daly rates for OA. In contrast, Cambodia had the lowest age-standardized prevalence and incidence rates, whereas Afghanistan had the lowest age-standardized Daly rates.

For knee OA, the age-standardized prevalence rates ranged from 8,336.68 to 21,432.96 per 100,000, the age-standardized incidence rates ranged from 666.11 to 1,481.88 per 100,000, and the age-standardized Daly rates ranged from 268.02 to 688.99 per 100,000. The Republic of Korea showed the highest values, whereas Tajikistan exhibited the lowest values.

For hip OA, the age-standardized prevalence rates ranged from 651.60 to 3,177.45 per 100,000, the age-standardized incidence rates ranged from 31.97 to 141.230 per 100,000, and the age-standardized Daly rates ranged from 20.91 to 99.30 per 100,000. The highest values were recorded in the United States of America, whereas the lowest values were seen in the Democratic People’s Republic of Korea.

For hand OA, the age-standardized prevalence rates ranged from 3,475.76 to 16,112.12 per 100,000, the age-standardized incidence rates ranged from 172.99 to 661.98 per 100,000, and the age-standardized Daly rates ranged from 110.3149031 to 510.6380988. The highest values were observed in Kazakhstan, while the lowest were observed in Burkina Faso.

**FIGURE 2:**
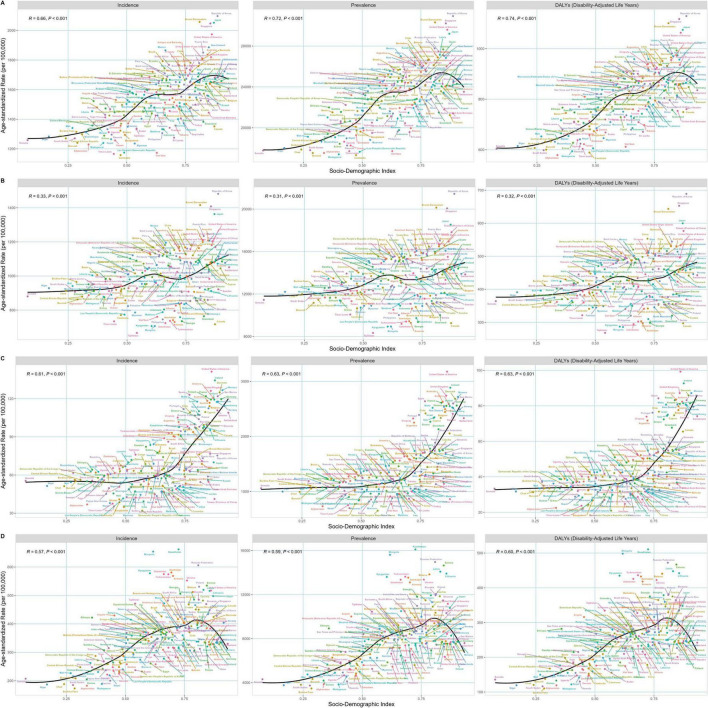
**(A)** The relationship between the SDI and the age-standardized incidence, prevalence, and Daly rates for total OA. **(B)** The relationship between the SDI and the age-standardized incidence, prevalence, and Daly rates for total knee OA. **(C)** The relationship between the SDI and the age-standardized incidence, prevalence, and Daly rates for total hip OA. **(D)** The relationship between the SDI and the age-standardized incidence, prevalence, and Daly rates for total hand OA.

We subsequently explored the association between SDI and the age-standardized incidence, prevalence, and Daly rates for total OA. The results revealed a significant positive correlation (Figure 2A). As SDI increased, the national average age-standardized incidence rates of OA initially rose. When SDI reached 0.625, the upward trend slowed and might even plateau, resuming an upward trajectory once SDI exceeds 0.75. Incidence rates decreased when SDI surpassed 0.8. Similarly, the age-standardized prevalence and Daly rates exhibited the same pattern of change.

Although the age-standardized incidence, prevalence, and Daly rates of overall OA was positively associated with SDI, differences existed among them. As shown in Figure 2B, the trend for knee OA was relatively gradual, indicating a slow increase in the age-standardized prevalence, incidence, and Daly rates of knee OA with rising SDI. In contrast, the hip OA initially showed a gradual increase, followed by a steep rise with increasing SDI (Figure 2C). The trend for hand OA was most similar to the total OA trend (Figure 2D).

### 3.5 Decomposition analysis of the OA burden

To evaluate the influence of three population-level factors-population growth, aging, and epidemiological shifts-on the epidemiology of OA over the past three decades (1990–2021), we performed a decomposition analysis of age-standardized Daly rates for each SDI quintile across the globe (Table 1).

**TABLE 1:** The decomposition analysis of Daly rates burden changes of OA by site, based on population growth, aging, and epidemiological changes from 1990 to 2021 at the global level and by SDI quintile.

Location	Overall difference	Chang due to population-level determinants (% contribution to the total change)		
		**Aging**	**Population**	**Epidemiological change**
**OA**				
Global	11834947.72	365652.21 (3.09)	10162162.37 (85.87)	1307133.14 (11.04)
Middle SDI	4270318.08	142522.77 (3.34)	3545140.74 (83.02)	582654.58 (13.64)
High SDI	2595527.3	203136.49 (7.83)	2110401.76 (81.31)	281989.05 (10.86)
High-middle SDI	2585491.05	80868.84 (3.13)	2202793.11 (85.2)	301829.11 (11.67)
Low SDI	554276.41	−9059.44 (−1.63)	493271.84 (88.99)	70064.01 (12.64)
Low-middle SDI	1821347.97	50674.54 (2.78)	1485836.49 (81.58)	284836.94 (15.64)
**Knee OA**				
Global	6547795.83	153643.65 (2.35)	5760666.79 (87.98)	633485.39 (9.67)
High SDI	1324449.11	91555.96 (6.91)	1110205.73 (83.82)	122687.42 (9.26)
High-middle SDI	1462651.55	28461.06 (1.95)	1190487.36 (81.39)	243703.13 (16.66)
Middle SDI	2414868.25	66079.58 (2.74)	2181298.56 (90.33)	167490.11 (6.94)
Low-middle SDI	1018934.3	23745.78 (2.33)	882103.76 (86.57)	113084.76 (11.1)
Low SDI	322919.54	−5847.65 (−1.81)	302245 (93.6)	26522.19 (8.21)
**Hip OA**				
Global	601599.55	29734 (4.94)	531292.29 (88.31)	40573.26 (6.74)
High SDI	204967.41	17483.79 (8.53)	158922.08 (77.54)	28561.54 (13.93)
High-middle SDI	127696.43	8235.12 (6.45)	112217.37 (87.88)	7243.95 (5.67)
Middle SDI	162589.91	7628.53 (4.69)	126503.37 (77.81)	28458.01 (17.5)
Low SDI	26286.06	−251.56 (−0.96)	23038.87 (87.65)	3498.75 (13.31)
Low-middle SDI	79587.82	2600.45 (3.27)	61161.16 (76.85)	15826.22 (19.89)
**Hand OA**				
Global	3591424.67	134507.92 (3.75)	2899829.62 (80.74)	557087.13 (15.51)
High SDI	847897.67	72305.9(8.53)	664043.51 (78.32)	111548.26 (13.16)
High-middle SDI	753038.47	32499.05 (4.32)	688980.12 (91.49)	31559.31 (4.19)
Middle SDI	1299440.75	47983.57 (3.69)	889266.76 (68.43)	362190.42 (27.87)
Low-middle SDI	540348.87	16571.95 (3.07)	379892.56 (70.31)	143884.35 (26.63)
Low SDI	147924.19	−1991.53 (−1.35)	112522.54 (76.07)	37393.18 (25.28)

**FIGURE 3:**
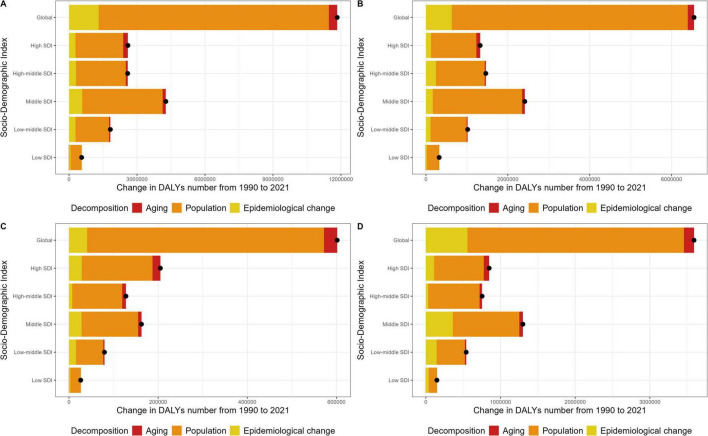
Changes in the Daly rates burden of OA by site, based on population growth, aging, and epidemiological changes from 1990 to 2021 at the global level and by SDI quintile. **(A)** Decomposition analysis of the determinants for total OA. **(B)** Decomposition analysis of the determinants for knee OA. **(C)** Decomposition analysis of the determinants for hip OA. **(D)** Decomposition analysis of the determinants for hand OA. The black dot represents the overall value of change contributed by all 3 determinants. For each determinant, the magnitude of a positive value indicates a corresponding increase in Daly rates attributed to the determinant; the magnitude of a negative value indicates a corresponding decrease in Daly rates attributed to the related determinant.

On a global scale, population growth accounted for 85.9% of the increase in OA age-standardized Daly rates, followed by epidemiological changes (11%) and population aging (3.1%). Significant rises in OA age-standardized Daly rates were noted across all SDI quintiles, with the most substantial increase occurring in the middle SDI quintile. The increases in the high-middle, high, and low-middle SDI quintiles were similar in magnitude, whereas the low SDI quintile exhibited the smallest increase (Figure 3A). The primary driver of the overall change in age-standardized Daly rates was population growth, which had the most substantial impact in the low SDI quintile (89%). In the other SDI quintiles, the contributions of population growth were also significant, exceeding 80% in each case. The effect of aging was most pronounced in the high SDI quintile (7.8%), diminishing progressively with lower SDI quintiles, and even turning negative (−1.6%) in the low SDI quintile. Epidemiological changes had the most significant influence in the low-middle SDI quintile (15.6%), with comparable effects observed across the remaining SDI quintiles (all above 10%).

For knee OA, 88% of the increase in age-standardized Daly rates were attributed to population growth, followed by epidemiological changes (9.7%) and aging (2.3%). Significant increase in OA age-standardized Daly rates were observed in all SDI quintiles, with the most significant increase in the middle SDI quintile, followed by comparable increases in the high-middle, high, and low-middle SDI quintiles. The increase in the low SDI quintile was the smallest (Figure 3B).

For hip OA, population growth accounted for 88.3% of the increase in age-standardized Daly rates. This was followed by epidemiological changes, which contributed 6.7%, and population aging, which accounted for 4.9%. The most significant increase in age-standardized Daly rates was observed in the high SDI quintile, followed by the middle, high-middle, low-middle, and low SDI quintiles (Figure 3C).

In the case of hand OA, population growth was the primary driver of the increase in age-standardized Daly rates, accounting for 80.7%. This was followed by contributions from epidemiological changes (15.5%) and population aging (3.7%). The most pronounced increase in age-standardized Daly rates was seen in the middle SDI quintile. Similar increases were observed in the high-middle, high, and low-middle SDI quintiles. In contrast, the low SDI quintile exhibited the smallest increase (Figure 3D).

### 3.6 Cross-national inequalities in the burden of OA

**FIGURE 4:**
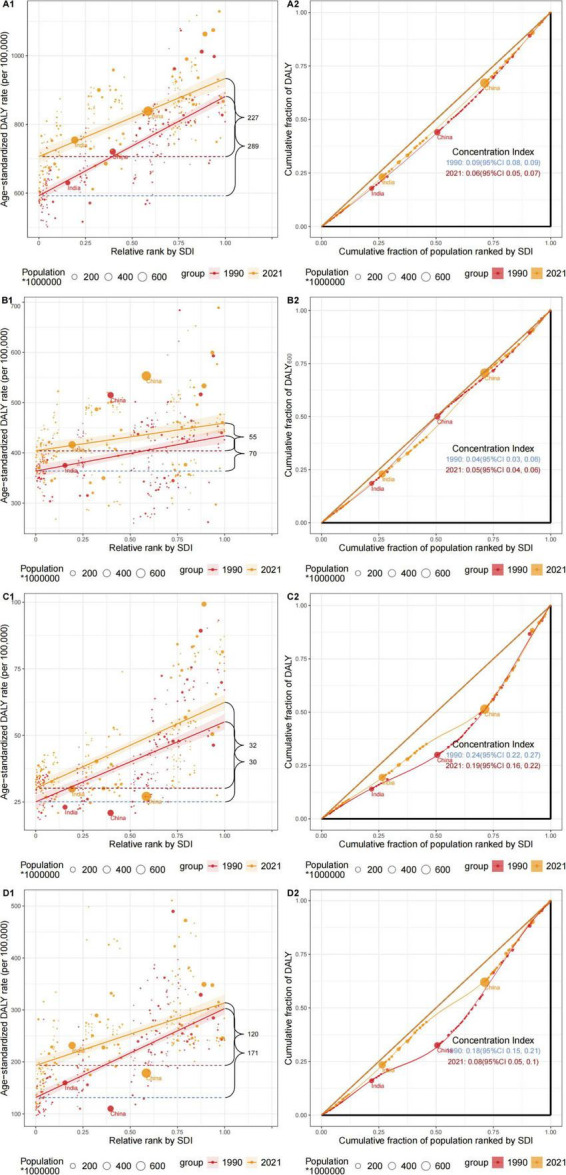
Health inequality regression curves and concentration curves for the Daly rates of OA worldwide, 1990 and 2021. **(A)** The health inequality regression curves and concentration curves for total OA. **(B)** The health inequality regression curves and concentration curves for knee OA. **(C)** The health inequality regression curves and concentration curves for hip OA. **(D)** The health inequality regression curves and concentration curves for hand OA.

On a global scale, notable disparities in the burden of total OA have been identified, both in terms of absolute and relative differences associated with the SDI. As shown in Figure 4A, age-standardized Daly rates exhibited a positive correlation with SDI levels, indicating that countries with higher SDI levels bear a greater OA-related health burden. In 1990, the inequality slope index for age-standardized Daly rates was 291.2 [95% CI (258-324.4)] per 100,000. This absolute inequality gap decreased to 229.6 [95% CI (196.5-262.6)] by 2021, reflecting a reduction in inequality in OA burden between countries over this period. While the concentration index, which measures relative inequality, was 0.09 [95% CI (0.08-0.09)] in 1990 and 0.06 [95% CI (0.05-0.07)] in 2021, indicating minimal change in relative inequality in OA burden during this period.

As illustrated in Figures 4B-D, age-standardized Daly rates for knee OA, hip OA, and hand OA were all positively correlated with SDI levels. However, the association between age-standardized Daly rates and SDI levels was weakest for knee OA, suggesting it is least affected by SDI levels.

However, the changes in inequality varied among different types of OA disease burdens. From the perspective of absolute inequality analysis, between 1990 and 2021, the slope index for knee OA and hand OA decreased from 71.5 [95% CI (43.2-99.8)] to 63.9 [95% CI (36.5-91.2)] and from 178.9 [95% CI (156.1-201.8)] to 123.1 [95% CI (96.2-150.1)], respectively. In contrast, the slope index for hip OA slightly increased from 33.6 [95% CI (28.53-38.6)] to 34.5 [95% CI (29.4-39.7)]. Over this period, the disparity in burdens of knee OA and hand OA between high-income and low-income countries decreased, while the inequality in hip OA burden grew.

In terms of relative inequality, the concentration index for knee OA rose slightly from 0.04 [95% CI: 0.03-0.06] to 0.05 [95% CI: 0.04-0.06]. Conversely, the concentration indices for hip osteoarthritis and hand OA declined, shifting from 0.24 [95% CI: 0.22-0.27] and 0.18 [95% CI: 0.15-0.21] in 1990 to 0.19 [95% CI: 0.16-0.22] and 0.07 [95% CI: 0.05-0.10], respectively.

## 4 Discussion

Based on the United Nations’ “World Population Prospects 2019”, the proportion of the elderly population is projected to increase from 9% in 2019 to 16% in 2025 and reach 1.5 billion by 2050 (23). As human life expectancy continues to increase, the incidence of joint trauma and inflammation is gradually rising. Concurrently, with advances in imaging diagnosis and an improved understanding of skeletal and joint diseases, the early detection rates of OA are gradually improving (24). Thus, the impact of OA on individuals, families, and society has significantly increased, posing a serious public health issue that threatens human physical and mental health (25). Previous epidemiological studies on OA have primarily been based on reports of the entire population or individual joints (26, 27), and the prevailing trends of OA across different sites have not yet been elucidated. Our study aims to investigate disease burden of OA in middle-aged and elderly populations, particularly in terms of site-specific trends over time. We provide a detailed, site-specific analysis of knee, hip, and hand OA. While previous research has examined OA burden globally, few studies have specifically analyzed the disease burden across different sites using the most recent data from the GBD Study 2021. The findings indicate that high SDI regions experience the highest OA burden, while low SDI countries show slower increases in age-standardized prevalence and Daly rates. We believe that this study contributes to the field by providing an understanding of OA trends in middle-aged and elderly populations.

Over the past 32 years, the overall burden of OA and its various sites has significantly increased. Actually, previous studies have utilized the GBD dataset to assess the burden of OA (22, 28). A key distinction is our study focus on middle-aged and elderly populations, as the condition is particularly impactful in this demographic. Moreover, the present study also focused on the sex difference and the burden of different types of OA. Among different types of OA, knee OA has the highest number of cases and incidence rates but does not exhibit the fastest rate of burden increase. In contrast, hand OA, which has the lowest burden, has shown a significantly higher growth rate compared to other OA sites over the past 32 years. The rapid increase in the burden of hand OA may be related to its higher prevalence among women (29, 30). With the gradual improvement in the status of women over the past three decades, there has been an increased focus on women’s health, leading to more accurate diagnoses of hand OA in women. Studies have confirmed that newly diagnosed cases of hand OA are more likely to be in younger women (10).

Compared with the relatively stable age-standardized rates, the overall case numbers of OA have significantly increased, indicating that the expansion of OA burden is primarily driven by changes in population levels. Decomposition analysis reveals that the increase in Daly number for OA at various sites is mainly attributed to population growth, particularly in hip OA and knee OA. Furthermore, this increase in burden is most evident in high SDI populations for hip OA and in middle SDI populations for knee and hand OA, while it is least pronounced in low SDI populations for all three types of OA. Notably, the epidemiological changes have led to a more substantial increase in the burden of hand OA compared to the other two forms, suggesting that changes in epidemiology more significantly affect the incidence of hand OA. This may be related to various factors, including alterations in risk factors, heightened awareness among women, and improvements in healthcare systems (31, 32).

From a regional perspective, the burden of OA has disproportionately concentrated in high SDI countries. The differences in disease burden between different SDI countries may be explained in part by the fact that areas with high SDI have more severe aging, increased rates of diagnosis, and increased rates of obesity, all of which are considered risk factors for OA (33). However, the positive correlation between OA burden and SDI levels has not consistently maintained the same slope. Initially, the burden of OA significantly increases with higher SDI levels, likely due to advancements in production technology and improved healthcare leading to population growth and increased detection of diseases in lower-middle-income regions. Nonetheless, this upward trend is constrained as SDI levels continue to rise, with decelerated population growth in higher-income countries leading to a more flattened trend, and even a decline in burdens within highly developed nations. The inequality concentration curve for OA burden indicates that, over time, the gap in OA burden between high-income and low-income countries is narrowing, with disproportionate knee OA burden in high SDI nations. On one hand, countries in high SDI regions have experienced industrialization, urbanization, and modernization, resulting in slowed population growth. On the other hand, the residents in these areas are more likely to have access to high-quality healthcare and medical services, thus mitigating to some extent the growth of disease burden in areas with high SDI (34). In addition, it can be related to regional differences in disease standards. With the prevalence of imaging diagnostics, the definition of OA continues to evolve, and the burden of disease in areas with low SDI continues to rise with new diagnoses in more potential patients, especially for previously understudied OA, such as the hand. Thus, there is a trend toward a certain degree of balance in the distribution of OA burden favoring highly developed countries.

Overall, this study is the first to comprehensively compare the disease burden and trends of different types of OA. Knee OA has the highest disease burden, while hand OA has the fastest growth rate over the past 31 years, and hip OA has the lowest disease burden and growth rate. The increasing trend of disease burden in different OA sites was different in the regional distribution and its relationship with SDI level. In the cross-national inequality analysis, the gap in the burden of knee and hand OA decreased between high-income and low-income countries, while the burden of hip OA increased slightly.

Effective measures to prevent the progression of OA disease burden must be proposed. First of all, although OA is commonly present in various joints of middle-aged and elderly people, people’s cognition and prevention of OA are limited. Common misconceptions mainly include the gender distribution of OA, risk factors, and insufficient cognition of physical therapy modalities, and low understanding of advanced treatment options such as injections (35). This highlights the need for targeted public health education to improve disease understanding and prevention measures. Secondly, the occurrence and development of OA in different parts are usually related to different population habits. Patients with knee arthritis have a higher risk of falls than those with non-knee osteoarthritis (36), hand arthritis may be associated with a state of sex hormone deficiency (30), and hip arthritis is thought to be associated with bone destruction due to various factors (37). Therefore, OA in different parts should take corresponding targeted preventive measures for different populations. Finally, although there are standard therapies for OA, their potential side effects cannot be ruled out and cannot provide complete treatment for patients, and it is urgent to find potential drug targets that are not difficult to develop targeted therapeutic drugs.

This research has several limitations: (1) This study is based on GBD 2021 study. Due to the potential for multiple joint OA to be redundantly measured within populations, it is challenging to accurately compare estimates across populations, which may lead to errors; (2) heterogeneity in OA definitions and measurement across regions but does not assess its potential impact on findings. For example, higher imaging rates in high SDI regions may inflate incidence estimates; (3) An analysis of OA risk factors has not been included and further investigations into factors such as gender, BMI, lifestyle, and work habits are necessary (22) to provide recommendations for future public prevention strategies.

## Data Availability

The original contributions presented in the study are included in the article/Supplementary material, further inquiries can be directed to the corresponding authors.

## Supplementary material

Supplementary Table 1 The prevalence, incidence, and Daly rates of OA globally and across varying SDI levels in 2021, and temporal trends from 1990 to 2021.

Supplementary Table 2 The prevalence of OA by site in 204 countries and territories in 2021 and time trends from 1900 to 2021.

Supplementary Table 3 The incidence of OA by site in 204 countries and territories in 2021 and time trends from 1900 to 2021.

Supplementary Table 4 The Daly rates of OA by site in 204 countries and territories in 2021 and time trends from 1900 to 2021.
