# Supplementary material for: Global, region and country burden of osteoarthritis at different sites in middle-aged and elderly populations from 1990 to 2021: a systematic analysis of the 2021 global burden of disease study
Source: Front Med (Lausanne). 2025 May 12;12:1567303. doi: 10.3389/fmed.2025.1567303 (PMC12104184; doi:10.3389/fmed.2025.1567303)
Supplement: Supplementary Table 5 — Supplementary methods. [file Table_5.docx]

**SDI computation**

SDI was originally constructed for GBD 2015 by using the Human Development Index (HDI) methodology, wherein a 0 to 1 index value was determined for each of the original three covariate inputs (TFR in ages 15 to 49 years, EDU15+, and LDI per capita) by using the observed minima and maxima over the estimation period to set the scales. In response to feedback from collaborators and the evolution of the GBD, we have refined the indicator with each GBD cycle. Beginning in GBD 2017, along with our expanded estimation of age‐specific fertility, we replaced TFR with TFU25 as one of the three component indices. The TFU25 provides a better measure of women’s status in society because it focuses on ages at which childbearing disrupts the pursuit of education and entrance into the workforce. In addition, we observed that in highly developed countries, the TFU25 has tended to decline consistently over time despite rebounds in TFR driven by increasing fertility at older ages.

Thus, for each covariate input, an index score of 0 represents the minimum level of each covariate input past which selected health outcomes can get no worse, and an indexscore of 1 represents the maximum level of each covariate input past which selected health outcomes cease to improve. As a composite, a ocation with an SDI of 0 would have a theoretical minimum level of sociodemographic development relevant to these health outcomes, and a location with an SDI of 1 (before multiplying by 100 for reporting) would have a theoretical maximum level of sociodemographic development relevant to these health outcomes.

We computed the index scores underlying SDI as follows:

$$I_{cly}=\max\left( \frac{C_{ly}-C_{low}}{C_{high}-C_{low}}, 0.05 \right)$$

Where:

$I_{cly}$is the index for covariate C, location l and year y and is equal to the difference between the value of this covariate in this location year and the lower limit of the covariate divided by the difference between the upper and lower limits for this covariate.

**The method of age standardization of data obtained from GBD.**

The formula for calculating ASR (per 100,000) can be expressed as follows.

$$ASR=\frac{\sum_{i=1}^{A} a_{i}w_{i}}{\sum_{i=1}^{A} w_{i}} \times100,000$$

Where ai refers to the ith age group and the number (or weight) (wi) of people in the same age subgroup i in the selected reference standard population.
